# Intraokularer Druckanstieg nach Vitrektomie – Applanationstonometrie nach Goldmann misst niedriger als dynamische Konturtonometrie

**DOI:** 10.1007/s00347-021-01443-z

**Published:** 2021-07-06

**Authors:** Sebastian Bäurle, Anja Viestenz, Berthold Seitz, Arne Viestenz

**Affiliations:** 1 Universitätsklinik und Poliklinik für Augenheilkunde, Universitätsklinikum Halle (Saale), Martin-Luther-Universität Halle-Wittenberg, Ernst-Grube-Str. 40, 06120 Halle, Deutschland; 2 Klinik für Augenheilkunde, Universitätsklinikum des Saarlandes (UKS), Kirrberger Straße 100, 66424 Homburg, Deutschland

**Keywords:** Pars-plana-Vitrektomie, Okuläre Hypertension, Erblindung, Gasexpansion, Winkelblock, Pars plana vitrectomy, Ocular hypertension, Angle closure, Blindness, Gas expansion

## Abstract

**Hintergrund:**

Das dynamische Konturtonometer PASCAL (DCT) ist ein digitales, der natürlichen Hornhautgeometrie angepasstes Kontakttonometer. Verglichen wurde das DCT mit dem Goldmann-Applanationstonometer (GAT).

**Methodik:**

In einer prospektiven Querschnittstudie wurden 100 Augen vor und nach Pars-plana-Vitrektomie (ppV) vergleichend mit dem GAT und DCT gemessen. Verwendete Endotamponaden waren verschiedene Gase und Silikonöle. Erfasst wurden der präoperative intraokulare Druck (IOD), postoperative Druckveränderungen und die Intertonometerdifferenz.

**Ergebnisse:**

Präoperativ lag der mittlere IOD mit dem GAT gemessen bei 15,8 ± 5,2 mm Hg und dem DCT bei 17,5 ± 5,9 mm Hg. Am ersten postoperativen Tag stieg bei Augen, die mit Gas versorgt wurden, der Druck im Mittel um 2,5 mm Hg (*p* = 0,035) an. Das DCT erfasste 18 Augen (19,1 %) mit einem postoperativen IOD von ≥ 25 mm Hg. Postoperativ maß das GAT den IOD im Schnitt 2,5 mm Hg niedriger und bei expansiver Gasendotamponade im Mittel um 3,0 mm Hg niedriger als das DCT. Bei IOD-Werten von postoperativ über 20 mm Hg lag das GAT im Mittel 4,7 mm Hg unter dem DCT. Zehn von 18 Augen mit IOD ≥ 25 mm Hg wurden mit dem GAT nicht als hypertensiv (≥ 25 mm Hg) erkannt. Bei 13 % maß das DCT am 1. postoperativen Tag einen um mindestens 6 mm Hg höheren IOD als das GAT. In einem Extremfall wurde der IOD mit dem GAT bei Gasendotamponade um 12 mm Hg unterschätzt.

**Schlussfolgerung:**

Postoperative Druckanstiege nach ppV sind gefürchtete Komplikationen und können zu irreversiblem Visusverlust führen. Abhängig von der verwendeten Endotamponade misst das GAT den IOD niedriger als das DCT – besonders bei Druckspitzen durch expansive Gase. Die postoperative IOD-Messung nach ppV ist bedeutend und die Messwerte von GAT und DCT können abweichen.

## Abstract (en)

**Background:**

The dynamic contour tonometer PASCAL (DCT) is a direct noninvasive digital tonometer matching the corneal contour. We compared the DCT with the Goldmann applanation tonometer (GAT).

**Methods:**

A total of 100 eyes were included in this prospective comparative clinical investigation and intraocular pressure (IOP) was measured with GAT and DCT before and after pars plana vitrectomy (ppV). Different intraocular endotamponades, such as gas and silicone oil were used. Preoperative IOP, changes in IOP postoperatively and intertonometer differences were measured.

**Results:**

The preoperative mean IOP was 15.8 ± 5.2 mm Hg measured with the GAT and 17.5 ± 5.9 mm Hg with DCT. On the first postoperative day, in eyes with gas endotamponade the mean IOP rise was 2.5 mm Hg (*p* = 0.035) and 18 eyes were measured with an IOP of ≥ 25 mm Hg (19.1%; DCT). Postoperative IOP measured by GAT was 2.5 mm Hg lower and in eyes with gas endotamponade the GAT measured the IOP 3.0 mm Hg lower than the DCT. In eyes with a postoperative IOP above 20 mm Hg, the GAT measured the IOP 4.7 mm Hg lower compared to the DCT. Out of 18 eyes 10 with IOP ≥ 25 mm Hg were not recognized as hypertensive (≥ 25 mm Hg) by GAT. In 13% of the eyes the DCT measured at least 6 mm Hg higher IOP levels than GAT on the first postoperative day. In one extreme case with gas endotamponade, GAT underestimated the IOP by 12 mm Hg compared to the DCT.

**Conclusion:**

The IOP spikes after ppV are a feared complication and can lead to irreversible visual loss. Depending on the endotamponade, GAT measures lower IOP than DCT, especially in IOP spikes caused by expansive gas endotamponade. Postoperative IOP measurement is important and readings between GAT and DCT can differ.

Postoperative intraokulare Druckanstiege gehören seit Beginn der modernen Netzhautchirurgie zu den häufigen postoperativen Komplikationen und können trotz eines komplikationslosen Operationsverlaufs zu irreversiblem Visusverlust führen. Das Goldmann-Applanationstonometer gilt als Goldstandard zur Messung des Intraokulardruckes und unterschätzt mitunter die Druckanstiege nach Vitrektomie.

## Einleitung und theoretischer Hintergrund

Der Intraokulardruck (IOD) ist ein entscheidender Risikofaktor für Glaukomerkrankungen. Bereits kurzfristige Druckspitzen können zu Nervenschädigungen mit irreversiblem Visusverlust führen. Durch einen hohen IOD kommt es u. a. zu einer ischämischen Nekrose von Axonen der retinalen Ganglienzellen, und übersteigt dieser den Perfusionsdruck der Zentralarterie, bricht die Versorgung der Retina ab. Extreme IOD-Anstiege können bereits nach 45 min zu ischämischen Schädigungen und Atrophie des N. opticus führen [1].

Das Messen des IOD gehört zur Standarddiagnostik, um eine okuläre Hypertension, eine manifeste Glaukomerkrankung oder kurzfristige postoperative IOD-Anstiege erkennen und behandeln zu können [25]. Noch heute wird der IOD in der gängigen Praxis mit dem Goldmann-Applanationstonometer (GAT) erfasst, welches als Goldstandard zur IOD-Bestimmung gilt. Das GAT wurde 1957 von Hans Goldmann vorgestellt und basiert als Applanationstonometer auf dem Imbert-Fick-Gesetz. Der IOD nach Goldmann entspricht einer Äquivalenzkraft, die benötigt wird, um die Hornhaut auf einer Fläche mit 3,06 mm Durchmesser zu applanieren. Als Resultat gilt die Goldmann-Applanationstonometrie idealerweise für Hornhäute mit einer Dicke von 520 μm [9].

Bereits Goldmann erwähnte nach Entwicklung des GAT, dass Faktoren wie die Hornhautdicke Einfluss auf die Applanationstonometrie haben müssen [9]. Nachfolgende Studien zeigten, dass das GAT in seiner Messgenauigkeit einer Beeinflussung durch Faktoren wie u. a. Hornhautdicke, Hydrationszustand und Hornhaut- und Sklerarigidität unterliegt [24, 29]. Neuere Tonometer, wie z. B. das dynamische Konturtonometer (DCT), setzen auf das Prinzip der direkten und der Hornhautkontur angepassten Messung des IOD [16]. Dadurch zeigt sich die Messgenauigkeit weniger beeinflusst durch Hornhautdicke oder weitere biomechanische Eigenschaften der Hornhaut [19]. Böhm et al. konnten eine Abweichung von < 1 mm Hg bei Kanülierung der Vorderkammer in vivo und der gleichzeitigen DCT-Messung nachweisen [4]. Das DCT zeigte im Vergleich zu einem manometrisch abgeleiteten IOD auch bei hydrierten Hornhäuten mit einem IOD bis 58 mm Hg eine geringe Abweichung und eignet sich daher zur genauen Erfassung des IOD und dem rechtzeitigen Erkennen von postoperativen Druckspitzen [20]. Besonders nach Pars-plana-Vitrektomie (ppV) ist der kurzfristige postoperative Druckanstieg eine häufige Komplikation [2, 25]. Insbesondere als Endotamponade verwendete expansive Gasgemische mit Schwefelhexafluorid (SF6), Perfluorethan (C2F6) und Perfluorpropan (C3F8) bergen die Gefahr von erheblichen IOD-Anstiegen. C3F8 hat in reiner Form mit ca. 4,2x den größten Expansionsfaktor und verbleibt bis zu 8 Wochen im Auge. SF6 expandiert mit einem Faktor von ca. 1,8x nicht so stark, erreicht dafür meist seine maximale Expansion bereits am 1. postoperativen Tag und verbleibt ca. 2 bis 6 Wochen im Auge [15].

Zusätzlich kann eine übermäßige Expansion des Gases zur Verschiebung des Iris-Linsen-Diaphragmas in Richtung Hornhaut führen. Dadurch wird der Kammerwinkel verschlossen, und es entsteht ein akutes Winkelblockglaukom. Dieses Phänomen kann bei Lagerungsincompliance, insbesondere Rückenlage des Patienten, beobachtet werden.

Ziel unserer Studie war es, den IOD-Verlauf nach vitreoretinalen Eingriffen unter Verwendung verschiedener Endotamponaden zu untersuchen und potenziell visusbedrohende postoperative Druckanstiege zu erkennen. Dabei wurde das etablierte GAT dem DCT gegenübergestellt.

## Patienten und Methodik

In dieser prospektiven Studie wurden 100 Patienten vor und nach ppV mit dem GAT und dem DCT gemessen. Die häufigsten Indikationen zur ppV waren: 29-mal epiretinale Gliose und 27-mal geplante Silikonölexplantation; 57 Patienten waren noch nicht vorvitrektomiert. In 67 Fällen bedurfte es einer temporär wirkenden Endotamponade, um langfristige Erfolge der operativen Therapie zu erzielen. Alle Gase wurden als nicht- oder gering-expansives Luft-Gas-Gemisch in das Auge gegeben. Silikonöle kamen als Polydimethylsiloxane (PDMS) in Kettenlänge und Viskosität von 2000 (*n* = 4) und 5700 (*n* = 5) Centistokes zum Einsatz.

Der IOD wurde sowohl präoperativ als auch postoperativ am Tag der ppV und am Folgetag mit dem GAT und DCT (*n* = 94) gemessen. Um eine Beeinflussung des Untersuchers zu vermeiden, wurde zuerst der IOD mit dem GAT in 2 Achsen (um 90° versetzt mit Berechnung des Mittelwerts) und anschließend digital mit dem DCT gemessen.

**Figure 1:**
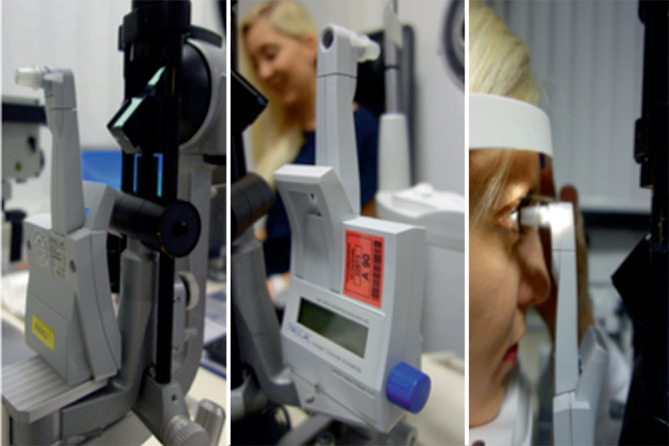

Das DCT PASCAL® (Ziemer Ophthalmic Systems AG; Port, Schweiz) setzt auf das Prinzip der konturangepassten Tonometrie mit einem der Hornhautkurvatur angepassten Tonometermessköpfchen mit eingelassenem piezoresistivem Drucksensor, welches insgesamt 500 Einzelmessungen durchführt. Die Messung des IOD ist direkt, nichtinvasiv und kontinuierlich. Nach einem akustischen Signal können auf einem LCD-Bildschirm der IOD, die okuläre Pulsamplitude (OPA) und eine Qualitätsstufe Q1–Q5 abgelesen werden [19]. Das DCT kann an eine herkömmliche Spaltlampe montiert werden (Abb. 1). Die Intra- und Interuntersuchervariabilität sind durch die einfache Handhabung, die automatische digitalisierte Berechnung und die Qualitätskontrolle gering und liegen unter der der Applanationstonometrie [17, 27].

Die statistische Datenanalyse erfolgte mithilfe des Statistik-Programms SPSS 20.0 (SPSS Inc.; Chicago, IL, USA). Die Messwerte wurden als Mittelwerte mit Standardabweichung angegeben. Der Zusammenhang zweier Variablen wurde durch den Korrelationswert nach Pearson ermittelt. Ein *p*-Wert von < 0,05 wurde als statistisch signifikant definiert.

## Ergebnisse

Präoperativ lag der mittlere IOD mit dem GAT gemessen bei 15,8 ± 5,3 mm Hg und mit dem DCT bei 17,5 ± 5,9 mm Hg. Die 57 nicht vorvitrektomierten Augen wiesen im Mittel den niedrigsten IOD auf (16,9 ± 4,8 mm Hg, gemessen mit dem DCT). Zusätzlich errechnete das DCT die okuläre Pulsamplitude (OPA). Diese lag im Mittel bei 2,5 mm Hg ± 1,3 mm Hg. Mit dem DCT gemessen, stieg der Druck am ersten postoperativen Tag im Schnitt um 1,2 mm Hg auf insgesamt 18,5 ± 8,4 mm Hg an. Mit dem GAT betrug der IOD am ersten postoperativen Tag im Mittel 15,9 ± 7,0 mm Hg (Tab. 1).

**Table 1:** 

	*N*	Minimum	Maximum	Mittelwert	Standardabweichung
GAT IOD	94	5	45,5	15,9	6,9
DCT IOD	94	7,7	56	18,5	8,4
DCT OPA	94	0,3	7	1,8	1,3

*OPA* okuläre Pulsamplitude, *GAT* Goldmann-Applanationstonometer, *DCT* dynamischen Konturtonometer

Die Messung mit dem DCT zeigte bei Augen, die mit Gas versorgt wurden, einen signifikanten postoperativen Druckanstieg innerhalb der ersten 24 h im Mittel um 2,5 mm Hg (*p* = 0,035) und am stärksten wenn SF6 eingesetzt wurde (+3,6 mm Hg) (Tab. 2). Der höchste mit dem GAT gemessene IOD lag bei 45,5 mm Hg, mit dem DCT wurden maximal 56 mm Hg gemessen (Delta: 10,5 mm Hg).

**Table 2:** 

	*N*	Differenz der Mittelwerte	95 %-Konfidenzintervall der Differenz		*p*-Wert
			Obere	Untere	
Gesamt	94	1,2	2,8	−0,4	0,15
Gas (Gesamt)	58	2,5	4,8	1,8	0,04
Gas (SF6)	22	3,6	8,4	−1,1	0,13
Gas (Gemisch)	36	1,6	3,9	−0,8	0,18
Silikonöl	9	3,6	8,1	−1,0	0,11

*SF6* Schwefelhexafluorid, *IOD* Intraokulardruck, *DCT* dynamischen Konturtonometer

Am ersten postoperativen Tag wurden 94 Patienten vergleichend mit dem DCT und GAT gemessen. Bei 6 Patienten gelang eine Messung mit dem DCT nicht. Mit dem GAT wurden bei diesen 6 Patienten hypotone IOD-Werte (< 5,0 mm Hg) gemessen. Während das GAT im Mittel präoperativ 1,8 mm Hg unter den Werten des DCT lag, maß das GAT postoperativ im Mittel 2,5 mm Hg niedriger als das DCT (*p* < 0,001). Bei Gasendotamponaden maß das GAT im Schnitt 2,9 mm Hg niedriger als das DCT (*p* < 0,001). Bei 12,7 % der gemessenen Augen maß das GAT den IOD um mindestens 6 mm Hg niedriger als das DCT (Tab. 3).

**Table 3:** 

Bereich	*N*	In %
∆IOD < 2,0	48	51,1
2,0 ≤ ∆IOD < 4,0	19	20,2
4,0 ≤ ∆IOD < 6,0	15	16,0
6,0 ≤ ∆IOD	12	12,7

*DCT* dynamischen Konturtonometer, *GAT* Goldmann-Applanationstonometer

**Figure 2:**
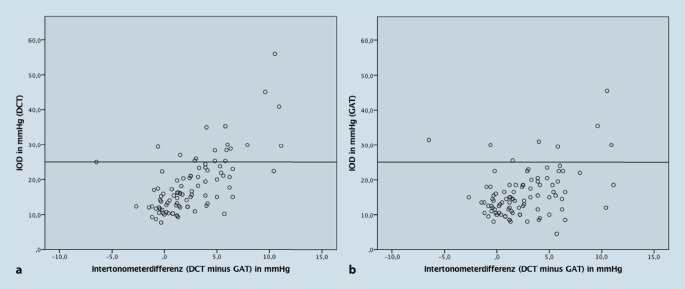

Insgesamt gab es eine positive Korrelation zwischen dem postoperativen IOD und der Intertonometerdifferenz. Das bedeutet, je höher der absolute IOD postoperativ war, desto größer war die Differenz zwischen den beiden Tonometern (IOD DCT r = 0,66, *p* < 0,001; IOD GAT r = 0,35, *p* = 0,01). Bei Augen, die postoperativ mit dem DCT gemessen, einen IOD von über 20 mm Hg aufwiesen, maß das GAT im Schnitt um 4,7 mm Hg niedriger. Sowohl mit dem DCT als auch mit dem GAT konnten einige Augen mit einem hypertensiven IOD von ≥ 25 mm Hg erfasst werden. Das DCT maß bei 18 Augen (19,1 %) postoperativ einen Wert von ≥ 25 mm Hg. Das GAT maß bei höheren IOD-Werten zunehmend niedrigere Werte als das DCT, weshalb nur 10 Augen mit einem IOD von ≥ 25 mm Hg postoperativ erfasst wurden (Abb. 2). Im Extremfall betrug die Intertonometerdifferenz 12 mm Hg, d. h. im Einzelfall maß das GAT den IOD 12 mm Hg niedriger als das DCT. Die zentrale Hornhautdicke hatte postoperativ im Mittel um 46 µm zugenommen und lag bei 601 µm (Spanne 468 µm–929 µm). Es zeigte sich keine signifikante Korrelation zwischen der Hornhautdicke und der Intertonometerdifferenz (r = −0,047; *p* = 0,657).

## Diskussion

### Druckanstieg nach ppV

Über die Problematik des postoperativen Druckanstiegs nach ppV wird seit Beginn der modernen Netzhautchirurgie berichtet. Erste Studien zeigten, dass bei 28 % der Augen nach ppV ein postoperativer Druckanstieg von mehr als 10 mm Hg beobachtet werden konnte [28]. Neuere Studien konnten zeigen, dass in den ersten 5–12 h 8,4 % der Patienten und am ersten Tag nach ppV 14,8 % einen IOD von über 29 mm Hg aufwiesen [2]. In unserer Studie konnten wir mit dem DCT einen moderaten IOD-Anstieg am ersten Tag nach ppV von ca. 1,2 mm Hg (+6,9 %) nachweisen. Jedoch zeigte sich bei der Verwendung von Gasendotamponaden ein höherer IOD-Anstieg um im Mittel 2,5 mm Hg (+14,3 %). Im Einzelfall beobachteten wir eine Druckspitze von 56 mm Hg (DCT); 18 Patienten (19,1 %) wiesen einen postoperativen IOD von ≥ 25 mm Hg auf (DCT). Der Mechanismus hinter IOD-Anstiegen kann vielfältig sein. Hauptfaktoren sind Gasexpansion, eine Trabekulitis und Druckanstiege durch Verlegung des Kammerwinkels durch Silikonöl, Erythrozyten, Fibrin in der Vorderkammer/Hinterkammer oder das Entstehen eines akuten Winkelblocks [5, 11]. Die postoperative Druckentwicklung kann durch Variieren der Konzentration der eingegebenen expansiven Gase und der Eingabemenge beeinflusst werden. Es bleiben dennoch schwer beeinflussbare Faktoren wie das individuelle Verhalten des Auges oder das Patientenverhalten in Bezug auf Lagerung und Compliance. Trotz komplikationslosem Operationsverlauf und dem physiologischen Verständnis über die Mechanismen hinter IOD-Anstiegen bleibt in manchen Fällen der postoperative Visusverlust ungeklärt. Unter anderem wurden postoperative Druckspitzen vermutet [14]. Die vorliegende Studie bestätigt, dass erhebliche Druckanstiege auch heutzutage zu den postoperativen Komplikationen nach ppV gehören. Das liegt u. a. am individuellen Vorbefund und der Reaktion des Auges, an der Gasexpansion sowie am postoperativen Verhalten der Patienten. Insbesondere regelmäßige postoperative Druckmessungen am Tag der ppV und darüber hinaus sowie eine optimale postoperative Lagerung der Patienten sind dabei essenziell.

### Intertonometerdifferenz zwischen DCT und GAT

Die beiden Tonometer GAT und DCT wurden in diversen Szenarien miteinander verglichen. Dabei maß das DCT im Vergleich zum GAT eher einen höheren Augendruck [3, 10, 12, 16, 27]. Kniestedt et al. zeigten eine hohe Messgenauigkeit des DCT. Im Vergleich zu einem manometrisch abgeleiteten IOD lag der systematische Messfehler bei unter 0,8 mm Hg [20]. Das DCT zeigt dabei eine geringere bis keine Beeinflussung der Messgenauigkeit im Vergleich zum GAT [7]. Der Hydrationszustand der Hornhaut wie z. B. bei postoperativen Hornhautödemen beeinflusst die Messgenauigkeit des DCT ebenfalls nicht signifikant [3, 13, 18]. In unserer Arbeit zeigte sich keine signifikante Korrelation zwischen der Hornhautdicke und der Intertonometerdifferenz.

Postoperativ wurde das DCT als zuverlässige Messmethode bestätigt. In Einzelfällen wurden große Differenzen zwischen DCT und GAT beobachtet [13, 22]. Sowohl nach Kataraktoperation als auch nach Keratoplastik misst das GAT signifikant niedrigere IOD-Werte als das DCT [12]. Viestenz et al. wiesen mit dem DCT einen um ca. 4 mm Hg höheren IOD als mit dem GAT an keratoplastizierten Augen nach [26]. Geht man von der beschriebenen Beeinflussbarkeit der Messgenauigkeit des GAT aus, kann angenommen werden, dass durch postoperative Veränderungen der Biomechanik der Hornhaut das GAT den IOD falsch zu niedrig misst [12].

Die vorliegende Arbeit gliedert sich in die Analysen oben aufgeführter Problematiken ein und untersucht den Zusammenhang zwischen dem Auftreten postoperativer Druckspitzen nach ppV und einer möglichen Unterschätzung des IOD durch das GAT. Konkret maß das GAT den IOD bei Gasendotamponaden durchschnittlich 2,9 mm Hg niedriger als das DCT.

Es zeigte sich ein positiver Zusammenhang zwischen dem absolut gemessenen IOD, sowohl mit dem DCT als auch mit dem GAT gemessen, und der Intertonometerdifferenz DCT minus GAT. Das heißt, dass bei höherem IOD das GAT im Vergleich zum DCT zunehmend niedriger maß. Dies hatte zur Konsequenz, dass von 18 Patienten, die mit dem DCT gemessen einen IOD von ≥ 25 mm Hg hatten, das GAT nur10 Patienten erfasste. Die Grenze von 25 mm Hg haben wir vordefiniert, und sie stellt keinen festen Marker dar. Das führt zu einem zu niedrig gemessenen Augeninnendruck bei 9 % der vitrektomierten Patienten und legt die Vermutung nahe, dass bei höherem IOD das GAT den IOD niedriger misst. Insbesondere bei Patienten mit zusätzlichen Risikofaktoren (arterielle Hypertonie, Blutdruck-Dippern) oder vorbestehendem Glaukomschaden kann eine IOD-Unterschätzung visusverschlechternde Folgen haben. Besonders in diesen Fällen ist eine engmaschige und exakte IOD-Bestimmung wichtig.

Mamas et al. haben ebenfalls das DCT vergleichend mit dem GAT nach ppV eingesetzt und wiesen eine Zunahme der Intertonometerdifferenz auf 3,1 mm Hg unter der Verwendung von SF6-Gas nach. In deren Studie maß das DCT den IOD 3,1 mm Hg niedriger. Nach Silikonölendotamponade lagen die Druckwerte mit dem GAT ebenfalls höher verglichen mit dem DCT. Demgegenüber zeigten am nicht vitrektomierten Auge DCT und GAT eine gute Übereinstimmung. Dies wirft die Frage auf, ob das DCT nach ppV den IOD-Anstieg unterschätzt [8, 22]. Kovacic et al. zeigten, dass nach Vitrektomie der IOD mit dem GAT gesunken ist (−2,0 mm Hg), wohingegen das DCT relativ höhere IOD-Werte maß (+0,7 mm Hg). Absolut maß das GAT postoperativ den IOD aber höher als das DCT (+1,2 mm Hg) [21]. Donaldson et al. konnten zeigen, dass das GAT postoperativ nach Eingabe von Endotamponaden den IOD im Schnitt 5,1 mm Hg niedriger maß. Ein signifikanter Unterschied zwischen den verschiedenen Endotamponaden (Luft, Gas und Silikonöl) konnte in ihrer Studie nicht nachgewiesen werden [6]. Da Vorstudien eine hohe Genauigkeit des DCT in verschiedenen Szenarien gezeigt haben und das GAT bei in der Biomechanik veränderten Hornhäuten den IOD falsch zu niedrig gemessen hat, liegt die Vermutung einer systematischen Unterschätzung seitens des GAT nahe, sofern eine Intertonometerdifferenz vorliegt [23]. In Tab. 3 wird die Intertonometerdifferenz mit Werten von ≥ 4 mm Hg bei 28,7 % und ≥ 6 mm Hg bei 12,7 % der Patienten gefunden. Der seltene Fall von einer Differenz von ≥ 10 mm Hg fanden wir bei 5 von 94 Patienten (5,3 %). Trotzdem ist die Messung mit dem GAT nicht als obsolet anzusehen und da in der täglichen Praxis jede Messung auch einer Messungenauigkeit unterliegt, benötigt es weiterhin klinischer Erfahrung, diese individuell zu beurteilen.

### Limitationen der Studie

Der Vergleich mit einem objektiven manometrisch intraokulär abgeleiteten IOD fehlt, was im postoperativen Setting zu invasiv für die Patienten gewesen wäre. Deshalb kann die vorliegende Arbeit nicht abschließend klären, ob das GAT den Augendruck wirklich unterschätzt, sondern zeigt in erster Linie, dass das GAT bei höherem IOD im Vergleich zum DCT niedrigere Werte misst. Weiterführende Untersuchungen, die das GAT und DCT bei höheren IOD-Werten gegenüberstellt, wären interessant, da in unserer Arbeit nur 18 Patienten einen IOD ≥ 25 mm Hg aufwiesen. Zur genaueren Erfassung der Zeitverläufe der IOD-Anstiege wäre eine engmaschigere vergleichende Messung mit exakter Zeitangabe (Stunden nach ppV) aufschlussreich gewesen. Dies ist im klinischen Alltag mit dem DCT praktisch nur schwer umzusetzen. Eine weiterführende Untersuchung könnte zeigen, ob insbesondere im Liegen die IOD-Werte mit GAT und/oder DCT ansteigen. Die Limitation unserer Studie beruht auf einer Messung des postoperativen IOD im Sitzen. Vereinzelt gelang außerdem die postoperative DCT-Messung bei hypotonen Augen nicht immer.

## Fazit für die Praxis

- Erhebliche Druckanstiege gehören auch in der heutigen Zeit zu den postoperativen Komplikationen nach Pars-plana-Vitrektomie (ppV) und sind schwer vorherzusagen.
- Besonders bei expansiven Gasendotamponaden ist das frühzeitige Erkennen von Druckspitzen wichtig. Regelmäßige Intraokulardruck(IOD)-Kontrollen (inklusive abendlicher Druckmessungen) unter stationären Bedingungen über einen Zeitraum von ca. 48 h postoperativ sind zu empfehlen.
- Dies empfehlen wir insbesondere bei Patienten mit arterieller Hypertonie, Blutdruck-Dippern und Patienten mit bestehendem Schaden der Papille/des N. opticus.
- Das dynamische Konturtonometer (DCT) stellt durch hohe Genauigkeit und Unabhängigkeit ein zuverlässiges Tonometersystem dar und kann ergänzend zum etablierten Goldmann-Applanationstonometer (GAT) visusbedrohende Druckanstiege rechtzeitig erkennen.
- Die postoperative IOD-Messung nach ppV ist bedeutend. Die Messwerte von GAT und DCT können abweichen.
